# Low T3 syndrome in heart failure: prevalence and its relationship with disease severity and ejection fraction

**DOI:** 10.1186/s12872-026-05973-9

**Published:** 2026-05-25

**Authors:** Celal Gezer, Hülya Yıldırım, Osman Mavis

**Affiliations:** 1 Department of Internal Medicine, Gaziosmanpaşa Training and Research Hospital, Istanbul, Turkey; 2 Department of Cardiology, Gaziosmanpaşa Training and Research Hospital, Istanbul, Turkey

**Keywords:** Heart failure, Low T3 syndrome, Disease severity, Ejection fraction, Thyroid hormones

## Abstract

**Background:**

Low T3 syndrome is a common hormonal alteration observed in patients with heart failure and has been associated with disease severity and adverse clinical outcomes. The prevalence of low T3 syndrome and its relationship with heart failure severity and ejection fraction phenotypes remain clinically relevant issues. This study aimed to evaluate the prevalence of low T3 syndrome in patients with heart failure and to investigate its association with disease severity and left ventricular ejection fraction.

**Methods:**

In this prospective observational study, 132 patients with chronic heart failure (81 males, 51 females) who were evaluated at a tertiary care hospital between June 2021 and November 2021 were consecutively enrolled. Patients with known thyroid disease, use of thyroid hormone therapy, positive anti-thyroid antibodies, severe anemia, malignancy, active inflammatory disease, or medications affecting thyroid function were excluded. Low T3 syndrome was defined as a serum total triiodothyronine level below 2.6 ng/L in the presence of normal thyroid-stimulating hormone concentrations. Patients were classified according to left ventricular ejection fraction as reduced (< 50%) or preserved (≥ 50%). Clinical characteristics, laboratory parameters, inflammatory markers, and hospitalization indicators were analyzed in relation to serum T3 levels and ejection fraction phenotypes.

**Results:**

Low T3 syndrome was detected in 60 patients (45.5%). Reduced ejection fraction was present in 82 patients (62.1%), while 50 patients (37.9%) had preserved ejection fraction. The prevalence of low T3 syndrome was significantly higher in patients with reduced ejection fraction compared to those with preserved ejection fraction. However, this association appeared less pronounced than its relationship with overall disease severity. Patients with low T3 syndrome demonstrated features of increased disease severity, including a non-significant trend toward longer hospital stays and worse clinical profiles. Lower T3 levels were associated with indicators of more advanced heart failure, whereas the relationship with ejection fraction phenotype was less pronounced.

**Conclusion:**

Low T3 syndrome is highly prevalent in patients with heart failure and is associated with increased disease severity. Its relationship with ejection fraction phenotype appears secondary to its association with clinical severity. Assessment of T3 levels may provide additional insight into the clinical status of patients with heart failure beyond conventional systolic function evaluation.

**Supplementary Information:**

The online version contains supplementary material available at 10.1186/s12872-026-05973-9.

## Introduction

Heart failure is a complex clinical syndrome associated with high morbidity, mortality, and recurrent hospitalizations worldwide [1]. Despite advances in contemporary therapies, disease progression and adverse outcomes remain common, indicating that heart failure is not solely a disorder of cardiac function but a systemic condition involving metabolic and hormonal alterations [2].

Thyroid hormones play a key role in cardiovascular regulation, influencing myocardial contractility, heart rate, vascular resistance, and energy metabolism [3]. In patients with chronic systemic illnesses, changes in thyroid hormone metabolism may occur in the absence of intrinsic thyroid disease. This condition, referred to as low T3 syndrome or non-thyroidal illness syndrome, is characterized by reduced circulating triiodothyronine levels with normal thyroid-stimulating hormone concentrations [4].

Low T3 syndrome has been frequently observed in patients with heart failure and is thought to reflect disease severity rather than primary thyroid dysfunction. Previous studies have reported associations between low T3 levels and worse clinical status and outcomes in heart failure populations [5]. Low T3 syndrome has been increasingly recognized as a marker of poor prognosis in patients with heart failure and has been associated with increased mortality, hospitalization, and disease severity in several studies [6–8]. However, the relationship between low T3 syndrome and left ventricular ejection fraction phenotype remains unclear, and its clinical relevance in this context continues to be debated. Despite previous evidence linking low T3 syndrome with heart failure, the extent to which it reflects overall disease severity beyond conventional parameters such as ejection fraction remains unclear. In particular, its relationship with systemic markers of disease burden, including inflammation and hospitalization characteristics, has not been fully elucidated. Unlike previous studies, the present study specifically aims to evaluate whether the relationship between low T3 syndrome and heart failure is more closely associated with overall disease severity rather than ejection fraction phenotype.

Therefore, the present study aimed to determine the prevalence of low T3 syndrome in patients with heart failure and to evaluate its relationship with disease severity and left ventricular ejection fraction.

## Methods

### Study design and population

This prospective observational study included patients diagnosed with chronic heart failure who were evaluated at a tertiary care hospital between June 2021 and November 2021. A total of 132 patients were consecutively enrolled during the study period. Patients younger than 18 years or older than 75 years were excluded. Transthoracic echocardiography was performed in all patients using a GE Vivid S60 ultrasound system (GE Healthcare, USA) equipped with a 2.5 MHz (GE 3 S) transducer. All echocardiographic evaluations were performed by a single experienced cardiologist to ensure consistency and minimize inter-observer variability. Left ventricular ejection fraction (LVEF) was calculated using the modified Simpson’s biplane method in accordance with current guideline recommendations [1]. Additional exclusion criteria included known thyroid disease, a history of thyroidectomy, use of thyroid hormone therapy, positive anti–thyroid peroxidase antibodies, severe anemia, active malignancy or inflammatory disease, and the use of medications known to interfere with thyroid function, such as amiodarone or systemic glucocorticoids.

Patients were enrolled both during hospitalization and outpatient follow-up visits. Blood samples for thyroid hormone measurements were obtained in the morning after an overnight fast at the time of clinical evaluation.

## Clinical and laboratory assessment

Demographic data, medical history, and clinical characteristics were recorded for all patients. Blood samples were obtained in the morning after an overnight fast for biochemical analyses. Thyroid function tests included serum total triiodothyronine, free thyroxine, and thyroid-stimulating hormone levels. Low T3 syndrome was defined as a total triiodothyronine level below 2.6 ng/L in the presence of normal thyroid-stimulating hormone concentrations. Total T3 levels were used in this study as they were routinely measured and consistently available for all patients in our clinical setting.

Disease severity was evaluated based on a combination of clinical and laboratory parameters, including hospitalization status, levels of inflammatory markers, and indicators of clinical deterioration. Based on these parameters, disease severity was categorized into stages (Stage 0–3), with higher stages reflecting more advanced disease. These parameters included NYHA functional class (III–IV indicating advanced disease), hospitalization status, duration of hospitalization, and inflammatory markers such as CRP (> 5 mg/L considered elevated). These parameters were selected based on their clinical relevance and prior use in the literature; however, no universally accepted definition exists, and this approach may introduce a degree of subjectivity. Due to the lack of a universally accepted definition for disease severity in this context, a composite assessment approach was adopted. This may introduce classification bias and affect the interpretation of the relationship between low T3 syndrome and disease severity. Advanced heart failure was defined based on the presence of NYHA class III–IV symptoms, need for hospitalization, prolonged hospital stays, and elevated inflammatory markers.

## Echocardiographic evaluation

Transthoracic echocardiography was performed according to standard clinical practice. Left ventricular ejection fraction was assessed using conventional methods. An ejection fraction cutoff of 50% was used to classify patients into reduced and preserved ejection fraction groups, in line with commonly used clinical practice. Patients were classified as having reduced ejection fraction when left ventricular ejection fraction was below 50% and preserved ejection fraction when it was 50% or higher.

### Statistical analysis

Continuous variables were expressed as mean ± standard deviation or median with interquartile range, as appropriate. Categorical variables were presented as frequencies and percentages. Comparisons between groups were performed using appropriate parametric or non-parametric tests. A *p* value of less than 0.05 was considered statistically significant. All statistical analyses were performed using SPSS software (version 22.0; IBM Corp., Armonk, NY, USA).

Variables with a *p*-value < 0.20 in univariate analyses were included in a multivariable logistic regression model to identify independent predictors of low T3 syndrome.

## Results

### Patient Characteristics

A total of 132 patients with chronic heart failure were included in the study, of whom 81 (61.4%) were male and 51 (38.6%) were female. The mean age of the study population was 65.1 ± 8.7 years. Baseline demographic features, comorbidities, and clinical characteristics of the patients are presented in Table 1.

**Table 1:** Baseline demographic and clinical characteristics

Variable	Value
Age, years	65.1 ± 8.7
Male sex, *n* (%)	81 (61.4)
Female sex, *n*(%)	51 (38.6)
Hyperte**n**sion, *n* (%)	132 (100%)
Diabetes mellitus, *n* (%)	68 (51.5%)
Coronary artery disease, *n* (%)	95 (71.9%)
Chronic kidney disease, *n* (%)	48 (36.3%)
Atrial fibrillation, *n* (%)	21 (15.9%)
NYHA class III–IV, *n* (%)	38 (28.8%)
CRP elevation (> 5 mg/L), *n*(%)	65 (49.2%)
Hemoglobin, g/dL	11.8 ± 2.2
Length of hospital stay, days	7(2–68)

*D*ata are presented as mean ± standard deviation or median (minimum–maximum), as appropriate. CRP elevation was defined as serum CRP > 5 mg/L. *NYHA* New York Heart Association, *CRP* C-reactive protein, Length of hospital stay was calculated among hospitalized patients only (*n* = 61).

### Prevalence of low T3 syndrome

**Fig. 1:**
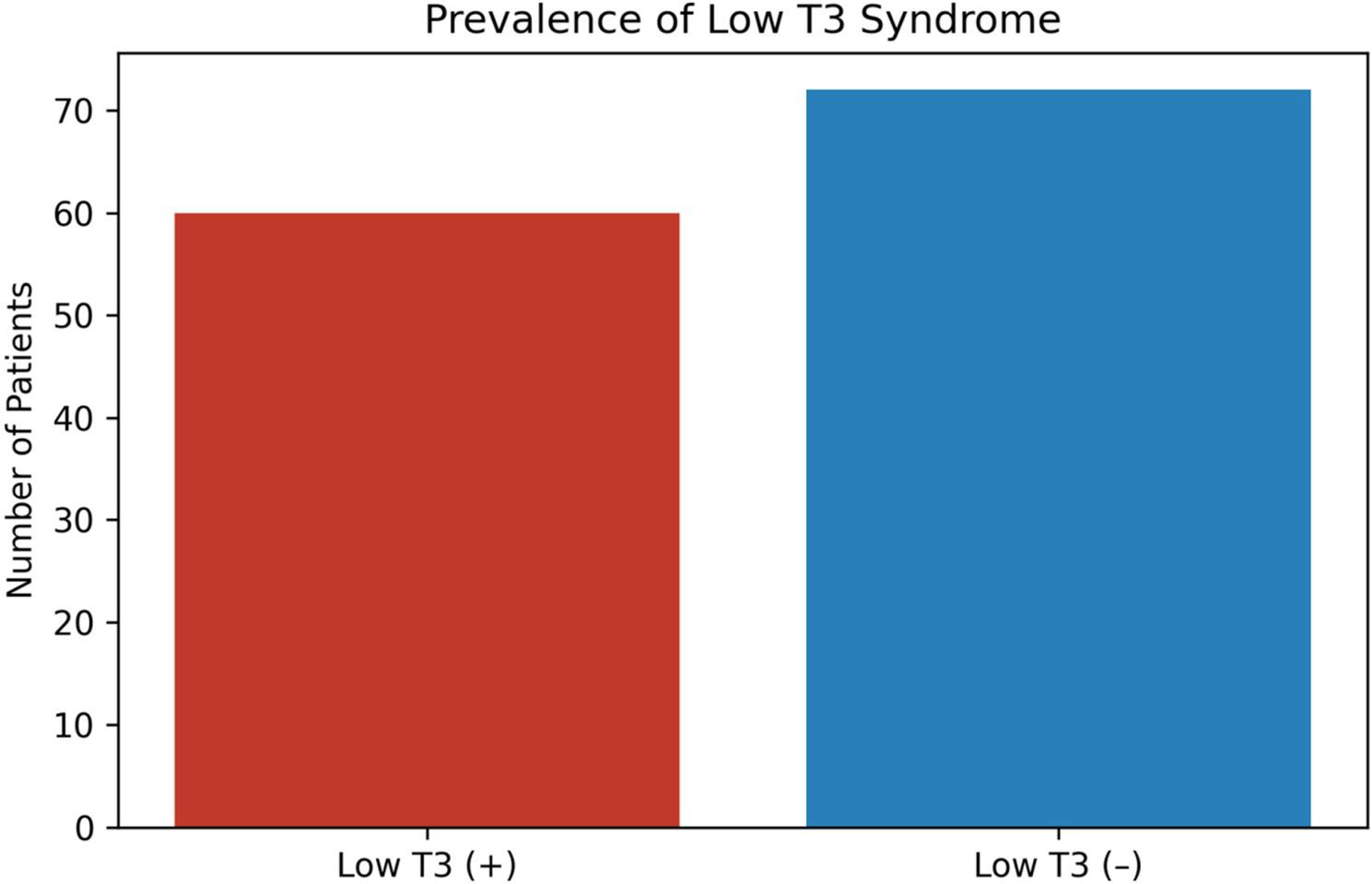
Prevalence of low T3 syndrome in the study population. Distribution of patients according to thyroid hormone status. Low T3 syndrome was defined as a serum total triiodothyronine level below 2.6 ng/L in the presence of normal thyroid-stimulating hormone concentrations

Low T3 syndrome was detected in 60 patients (45.5%). The overall prevalence of low T3 syndrome in the study population is illustrated in Fig. 1. The distribution of patients according to thyroid hormone status and its relationship with clinical and laboratory parameters is summarized in Table 2. Patients with low T3 syndrome demonstrated features of increased disease severity, including higher inflammatory markers and a non-significant trend toward longer duration of hospitalization.

**Table 2:** Prevalence of low T3 syndrome and its relationship with disease severity

Variable	Low T3 (+) (*n* = 60)	Low T3 (–) (*n* = 72)	*p* value
Length of hospital stay, days†	12.73 ± 13.2	8.76 ± 5.4	0.589
Hospitalization, *n* (%)	25 (41.7)	36 (50.0)	0.165
CRP elevation (> 5 mg/L), *n* (%)	37 (61.7)	28 (38.9)	0.015
NYHA class III–IV, *n* (%)	23 (38.3)	15 (20.8)	0.044

Data are presented as number and percentage or median (minimum–maximum), as appropriate. Low T3 syndrome was defined as a serum total triiodothyronine level < 2.6 ng/L with normal thyroid-stimulating hormone levels. Comparisons between groups were performed using the chi-square test or Mann–Whitney U test, as appropriate. A *p* value < 0.05 was considered statistically significant

†Length of hospital stay was calculated among hospitalized patients only

Distribution of patients according to thyroid hormone status and its association with clinical and laboratory indicators of heart failure severity. Low T3 syndrome was defined as a total triiodothyronine level below 2.6 ng/L in the presence of normal thyroid-stimulating hormone levels

### Ejection fraction phenotypes

**Fig. 2:**
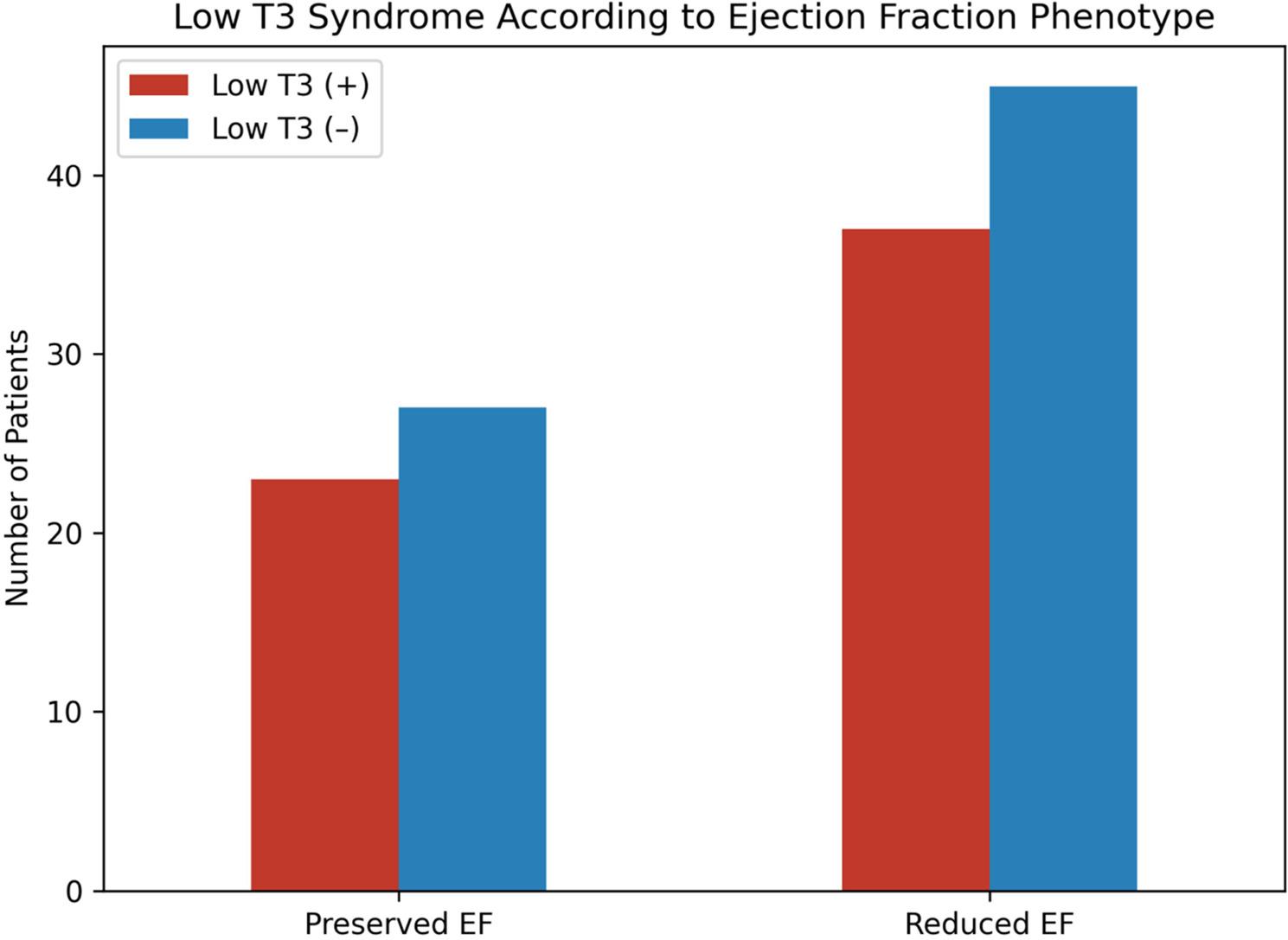
Distribution of low T3 syndrome according to ejection fraction phenotype. Comparison of the prevalence of low T3 syndrome between patients with reduced and preserved left ventricular ejection fraction. A significant difference was observed between groups

Among the study population, 82 patients (62.1%) had reduced ejection fraction, while 50 patients (37.9%) had preserved ejection fraction. The distribution of low T3 syndrome according to ejection fraction phenotype is shown in Fig. 2. The relationship between ejection fraction phenotypes, inflammatory markers, and hospitalization characteristics is presented in Table 3. The prevalence of low T3 syndrome was significantly higher in patients with reduced LVEF compared to those with preserved LVEF (57.3% vs. 26.0%, *p* < 0.001).

**Table 3:** Ejection fraction phenotypes and clinical associations

Variable	Reduced EF (*n* = 82)	Preserved EF (*n* = 50)	*p* value
Male sex, *n* (%)	60 (73.2)	21 (42.0)	**< 0.001**
Female sex, *n* (%)	22 (26.8)	29 (58.0)	
NYHA class II, *n* (%)	11 (13.4)	19 (38.0)	**0.002**
NYHA class III, *n* (%)	43 (52.4)	25 (50.0)	
NYHA class IV, *n* (%)	28 (34.1)	6 (12.0)	
Low T3 syndrome, *n* (%)	47 (57.3)	13 (26.0)	**< 0.001**
CRP elevation, *n* (%)	51 (62.2)	21 (42.0)	**0.024**
Anemia, *n* (%)	47 (57.3)	30 (60.0)	0.762
Hospitalization, *n* (%)	43 (52.4)	20 (40.0)	0.165

Data are presented as number and percentage. *EF* ejection fraction, *CRP* C-reactive protein, *NYHA* New York Heart Association, Low T3 syndrome was defined as a serum total triiodothyronine level < 2.6 ng/L with normal thyroid-stimulating hormone levels. Comparisons between groups were performed using the chi-square test. A *p* value < 0.05 was considered statistically significant

Comparison of clinical characteristics, inflammatory markers, and hospitalization status according to left ventricular ejection fraction phenotype. Patients were classified as having reduced ejection fraction (< 50%) or preserved ejection fraction (≥ 50%). Categorical variables are presented as number and percentage, and continuous variables as mean ± standard deviation or median (interquartile range)

Bold values indicate statistically significant diffrerences (*p *< 0.05)

### Relationship between thyroid hormones, disease severity, and hospitalization

Lower serum T3 levels were associated with more advanced heart failure and were also linked to a non-significant trend toward longer hospital stay. In addition, low T3 syndrome was more prevalent in patients with reduced ejection fraction. However, the association between low T3 syndrome and overall disease severity appeared more pronounced than its relationship with ejection fraction alone. These findings suggest that low T3 syndrome reflects the overall clinical burden of heart failure and is not solely dependent on systolic function.

Multivariable logistic regression analysis was performed to identify independent predictors of low T3 syndrome. The overall model was statistically significant (χ² = 59.642, *p* < 0.001) and demonstrated good explanatory power (Nagelkerke R² = 0.486), with an overall correct classification rate of 78.8%.

Disease stage was identified as an independent predictor of low T3 syndrome, while albumin status showed a trend toward significance. Patients in stage 0 had a higher likelihood of having normal T3 levels compared to those in stage 3 (OR: 4.651, *p* = 0.044).

Albumin status showed a trend toward significance, with patients with normal albumin levels more likely to have normal T3 levels compared to those with hypoalbuminemia (OR: 2.899, 95% CI: 0.935–8.995, *p* = 0.065). The results of the multivariable logistic regression analysis are presented in Table 4. These results support the association between low T3 syndrome and heart failure severity after adjustment for potential confounders.

**Table 4:** **–** Multivariable logistic regression analysis of factors associated with low T3 syndrome

Variable	OR (95% Cl)	*p* value
Albumin (normal vs. hypoalbuminemia)	2.899 (0.935–8.995)	0.065
Disease stage (0 vs. 3)	4.651 (0.72–58.8)	0.044

Variables included in the multivariable model were selected based on univariate comparisons (*p* < 0.20). Reference categories were hypoalbuminemia for albumin and stage 3 for disease severity

*OR* odds ratio, *CI* confidence interval

## Discussion

### Principal findings

In this study, low T3 syndrome was found to be highly prevalent among patients with chronic heart failure. The main findings can be summarized as follows: low T3 syndrome was common in the study population and was associated with markers of increased disease severity, with a non-significant trend toward longer hospitalization. Low T3 syndrome in heart failure is thought to reflect an adaptive but ultimately maladaptive response involving reduced peripheral conversion of T4 to T3, increased inflammatory cytokine activity, and altered deiodinase function. This may contribute to impaired myocardial energy utilization, reduced contractility, and worsening clinical status [5, 9]. Low T3 syndrome was more prevalent in patients with reduced ejection fraction; however, this difference appeared secondary to its stronger association with overall disease severity, suggesting that it reflects global disease burden rather than systolic function alone.

### Low T3 syndrome and heart failure severity

Alterations in thyroid hormone metabolism are well recognized in chronic systemic diseases, including heart failure. Reduced circulating T3 levels are thought to represent an adaptive response to chronic illness, aiming to reduce energy expenditure during periods of metabolic stress [9]. In heart failure, this adaptive response may become maladaptive, as thyroid hormones play a critical role in maintaining myocardial contractility, vascular tone, and cellular metabolism.

Previous hospitalizations have been shown to be an important marker of disease severity and are associated with worse prognosis in patients with heart failure. In this context, Perea-Armijo et al. demonstrated that the number of hospital admissions is strongly related to disease progression and outcomes [10].

Previous studies have consistently demonstrated an association between low T3 levels and advanced heart failure severity, poorer functional status, and adverse clinical outcomes, with several meta-analyses confirming its independent prognostic significance across different heart failure populations [6–8, 11].

Our findings support this concept, as patients with low T3 syndrome exhibited markers of more severe disease and were associated with a non-significant trend toward longer hospital stay. Our findings are consistent with previous studies demonstrating an association between low T3 syndrome and worse clinical outcomes in heart failure. However, our results further suggest that this relationship is more closely linked to overall disease burden rather than systolic function alone.

### Relationship between low T3 syndrome and ejection fraction phenotype

The relationship between low T3 syndrome and left ventricular ejection fraction remains controversial. While some reports suggest that low T3 syndrome is more prevalent in patients with reduced ejection fraction, other studies have shown comparable frequencies across different ejection fraction phenotypes [8, 12]. In our cohort, the prevalence of low T3 syndrome was significantly higher in patients with reduced ejection fraction compared to those with preserved ejection fraction. However, the association between low T3 syndrome and overall disease severity appeared more prominent than its relationship with ejection fraction alone.

This finding highlights the limitations of ejection fraction–based classification in capturing the systemic and metabolic dimensions of heart failure. Heart failure with preserved ejection fraction, in particular, is increasingly recognized as a heterogeneous syndrome driven by comorbidities and systemic inflammation rather than isolated myocardial systolic dysfunction [13].

### Clinical implications

From a clinical perspective, the presence of low T3 syndrome in patients with heart failure may provide additional information regarding disease severity and prognosis. Although routine thyroid hormone replacement cannot be recommended based on current evidence, assessment of T3 levels may help identify patients with higher clinical risk [14–16]. These findings suggest that low T3 syndrome may serve as a simple and clinically accessible marker of disease severity, aiding in risk stratification while reflecting systemic illness rather than an isolated endocrine abnormality.

### Study limitations

One important limitation of this study is the absence of a standardized and validated definition of disease severity in this context. The composite severity classification used in our analysis was based on clinically relevant parameters, including NYHA functional class, hospitalization status, duration of hospitalization, and inflammatory markers; however, this approach may introduce subjectivity and potential classification bias.

Given that disease severity represents a central variable in our analysis, this limitation should be carefully considered when interpreting the strength and independence of the observed associations between low T3 syndrome and clinical status. In addition, residual confounding cannot be completely excluded despite multivariable analysis.

Although the overall sample size of the study was moderate, subgroup analyses—particularly those based on ejection fraction phenotypes—may have been underpowered to detect smaller differences between groups. This limitation is especially relevant when interpreting the relatively weaker association observed between low T3 syndrome and ejection fraction compared to overall disease severity.

Another important limitation is the absence of hard clinical outcomes such as mortality or rehospitalization. Therefore, the findings of this study should be interpreted as associations with disease severity rather than as indicators of prognostic outcomes. The lack of longitudinal outcome data limits the ability to draw conclusions regarding the predictive or prognostic value of low T3 syndrome in this population.

In addition, the classification of ejection fraction using a single cutoff value of 50% without separate evaluation of the mid-range ejection fraction (HFmrEF) category may represent a limitation. Although this simplified classification was adopted to maintain statistical robustness and avoid further reduction in subgroup sample sizes, it may have limited the ability to detect potential differences in thyroid hormone profiles across more refined ejection fraction phenotypes. As a result, the present study does not allow for a detailed evaluation of the relationship between low T3 syndrome and specific ejection fraction subgroups such as HFmrEF.

This supports the concept that low T3 syndrome reflects the systemic burden of disease, including inflammatory activity and clinical instability, rather than being solely a marker of impaired ventricular function.

Furthermore, due to the lack of mortality data, we were unable to evaluate the association between low T3 syndrome and mortality outcomes, which represents an important limitation. Finally, the observational design of the study limits the ability to establish a causal relationship between low T3 syndrome and heart failure severity.

Thyroid hormone levels were assessed at a single time point, which may not reflect dynamic changes during disease progression or acute decompensation. The lack of serial measurements limits the ability to evaluate temporal variations in T3 levels and their potential relationship with changes in clinical status. Future longitudinal studies with larger and more diverse cohorts are needed to validate the role of low T3 syndrome as a marker of disease burden and to clarify its relationship with hard clinical outcomes in heart failure.

## Conclusion

Low T3 syndrome is a common finding in patients with chronic heart failure and is associated with indicators of greater disease severity, with a non-significant trend toward longer hospitalization. Although it is more frequently observed in patients with reduced ejection fraction, its relationship with overall clinical severity appears more prominent than its association with ejection fraction phenotype alone. Evaluation of thyroid hormone alterations may provide additional insight into the systemic nature of heart failure and help identify patients with a higher clinical burden. 

## Supplementary Information

Supplementary Material 1.

## Data Availability

The datasets used and/or analyzed during the current study are available from the corresponding author on reasonable request.
